# *catena*-Poly[[[tetra­kis(μ-2-butenoato)dicopper(II)]-μ-2-butenoato-[diaqua­(2-butenoato)holmium(III)]-di-μ-2-butenoato-[diaqua­(2-butenoato)holmium(III)]-μ-2-butenoato] trihydrate]

**DOI:** 10.1107/S1600536808034296

**Published:** 2008-10-25

**Authors:** Mireille Perec, Maria Teresa Garland, Ricardo Baggio

**Affiliations:** a Departamento de Química Inorgánica, Analítica y Química Física, INQUIMAE, Facultad de Ciencias Exactas y Naturales, Universidad de Buenos Aires, Ciudad Universitaria, Pabellón II, 1428 Buenos Aires, Argentina; b Departamento de Física, Facultad de Ciencias Físicas y Matemáticas and CIMAT, Universidad de Chile, Santiago de Chile, Chile; c Departamento de Física, Centro Atómico Constituyentes, Comisión Nacional de Energía Atómica., Buenos Aires, Argentina

## Abstract

The title compound {[Cu_2_Ho_2_(C_4_H_5_O_2_)_10_(H_2_O)_4_]·3H_2_O}_*n*_, is a one-dimensional 3*d*/4*f* organic–inorganic hybrid complex, the Ho^III^ member of the isotypic lanthanoid series with *Ln* = Gd^III^, Er^III^ and Y^III^. The structure shows an alternation of Cu_2_ and Ho_2_ dinuclear units bridged by the ligands and hydrogen bonds only. The chains are composed of Cu_2_ classical dinuclear η^1^:η^1^:μ_2_ fourfold bridges [Cu⋯Cu = 2.6417 (9) Å] and of Ho_2_ units bridged by two η^2^:η^1^:μ_2_ carboxyl­ate units. This results in distorted square-based pyramidal CuO_5_ units and irregular HoO_9_ units. The alternating Cu_2_ and Ho_2_ units are bridged into linear arrays along the *a* axis by a set of one η^2^:η^1^:μ_2_ carboxyl­ate O atom and two hydrogen bonds with Cu⋯Ho separations of 4.4883 (10) and 4.5086 (10) Å. The distance between adjacent chains, as calculated by the closest and furthest distances between two chains, covers the range 10–14 Å. The H atoms of the water mol­ecules could not be located, but the O⋯O separations for these species suggest the presence of O—H⋯O hydrogen bonds.

## Related literature

For related structures, see: Benelli & Gatteschi (2002 ▶); Kutlu *et al.* (1997 ▶); Legendziewicz *et al.* (2000 ▶). For the isotypic family, see: Calvo *et al.* (2008 ▶). For related literature, see: van Niekerk & Schoening (1953 ▶). For bond-length data, see: Allen (2002 ▶).
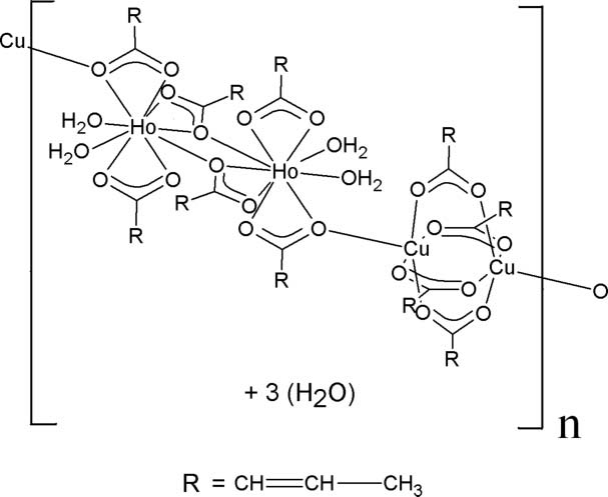


## Experimental

### 

#### Crystal data

[Cu_2_Ho_2_(C_4_H_5_O_2_)_10_(H_2_O)_4_]·3H_2_O
*M* *_r_* = 1433.85
Monoclinic, 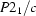

*a* = 13.8856 (17) Å
*b* = 22.078 (3) Å
*c* = 19.846 (3) Å
β = 107.152 (2)°
*V* = 5813.5 (14) Å^3^
*Z* = 4
Mo *K*α radiation
μ = 3.49 mm^−1^
*T* = 291 (2) K
0.24 × 0.10 × 0.08 mm

#### Data collection

Bruker SMART CCD area-detector diffractometer
Absorption correction: multi-scan (*SADABS*; Bruker, 2002 ▶) *T*_min_ = 0.62, *T*_max_ = 0.76
34338 measured reflections
12977 independent reflections
9482 reflections with *I* > 2σ(*I*)
*R*_int_ = 0.043

#### Refinement

*R*[*F* ^2^ > 2σ(*F* ^2^)] = 0.040
*wR*(*F* ^2^) = 0.085
*S* = 0.93
12977 reflections
653 parameters
30 restraints
H-atom parameters constrained
Δρ_max_ = 1.23 e Å^−3^
Δρ_min_ = −1.02 e Å^−3^

### 

Data collection: *SMART-NT* (Bruker, 2001 ▶); cell refinement: *SAINT-NT* (Bruker, 2002 ▶); data reduction: *SAINT-NT*; program(s) used to solve structure: *SHELXS97* (Sheldrick, 2008 ▶); program(s) used to refine structure: *SHELXL97* (Sheldrick, 2008 ▶); molecular graphics: *SHELXTL* (Sheldrick, 2008 ▶); software used to prepare material for publication: *SHELXTL* and *PLATON* (Spek (2003 ▶).

## Supplementary Material

Crystal structure: contains datablocks I, global. DOI: 10.1107/S1600536808034296/hb2821sup1.cif

Structure factors: contains datablocks I. DOI: 10.1107/S1600536808034296/hb2821Isup2.hkl

Additional supplementary materials:  crystallographic information; 3D view; checkCIF report

## Figures and Tables

**Table 1:** Selected bond lengths (Å)

Ho1—O17	2.322 (3)
Ho1—O1*W*	2.323 (3)
Ho1—O18	2.356 (3)
Ho1—O10	2.362 (4)
Ho1—O29	2.365 (3)
Ho1—O2*W*	2.386 (3)
Ho1—O20	2.418 (3)
Ho1—O19	2.619 (3)
Ho1—O28	2.789 (4)
Ho2—O28	2.316 (3)
Ho2—O4*W*	2.322 (3)
Ho2—O26	2.357 (3)
Ho2—O3*W*	2.368 (3)
Ho2—O25	2.382 (3)
Ho2—O27	2.392 (3)
Ho2—O15	2.443 (3)
Ho2—O16	2.635 (3)
Ho2—O17	2.642 (3)
Cu1—O12	1.939 (3)
Cu1—O13	1.949 (4)
Cu1—O14	1.961 (3)
Cu1—O11	1.978 (3)
Cu1—O16^i^	2.215 (3)
Cu2—O21	1.938 (4)
Cu2—O23	1.968 (3)
Cu2—O24	1.971 (3)
Cu2—O22	1.989 (3)
Cu2—O19	2.178 (3)

Symmetry code: (i) 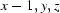.

**Table 2:** Short O*W*⋯O contacts (< 3.00 Å) attributable to hydrogen bonding

O*W*⋯O	*d* (Å)		O*W*⋯O	*d* (Å)
O1*W*⋯O18	2.948 (5)		O3*W*⋯O28	2.957 (5)
O1*W*⋯O19	2.815 (5)		O4*W*⋯O14^i^	2.645 (5)
O1*W*⋯O20	2.895 (5)		O4*W*⋯O15	2.853 (5)
O1*W*⋯O22	2.763 (5)		O4*W*⋯O16	2.839 (4)
O1*W*⋯O5*W*	2.632 (5)		O4*W*⋯O27	2.854 (5)
O2*W*⋯O19	2.900 (5)		O4*W*⋯O7*W*^ii^	2.703 (5)
O2*W*⋯O23	2.777 (5)		O5*W*⋯O27^iii^	2.848 (5)
O2*W*⋯O25	2.734 (5)		O5*W*⋯O6*W*	2.800 (5)
O2*W*⋯O28	2.905 (5)		O6*W*⋯O15^iii^	2.788 (5)
O3*W*⋯O10	2.675 (5)		O6*W*⋯O26^iv^	2.858 (5)
O3*W*⋯O11^i^	2.766 (5)		O6*W*⋯O7*W*	2.867 (5)

Symmetry codes: (i) 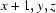; (ii) 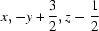; (iii) 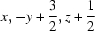; (iv) 
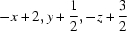
.

**Fig. 1.**
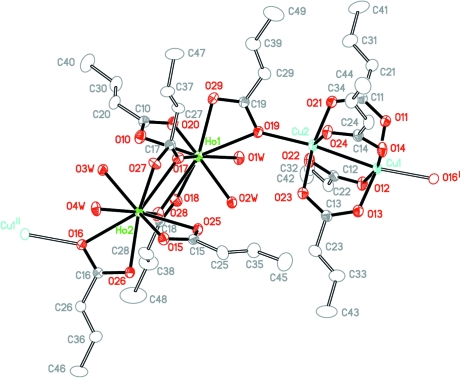
Molecular view of both dimers, with displacement ellipsoids at a 30% level. H atoms omitted, and carbon atoms not in the carboxylate groups drawn in empty ellipsoids and bonds, for clarity.

**Fig. 2.**
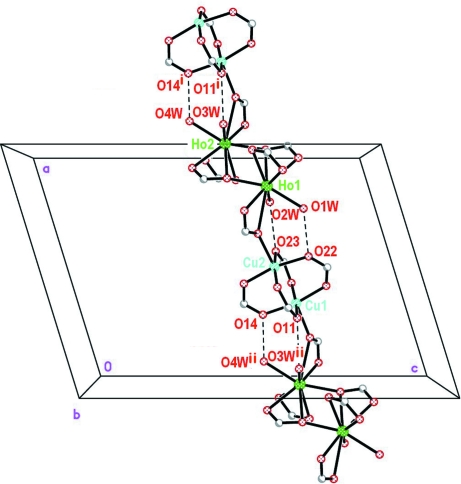
Schematic view of a chain running along [100]. Possible intrachain H-bonds drawn in broken lines. All H atoms and those carbon atoms not in the carboxylate groups, omitted for clarity. Symmetry codes: (i) 1 + x, y, z; (ii): -1 + x, y, z

## supplementary crystallographic information

### Comment

In the field of molecule-based magnetic materials of 3 d and 4f metals with multicarboxylate bridging ligands, a variety of two-dimensional and three-dimensional original structures with interesting magnetic and spectroscopic properties have been discovered (Benelli and Gatteschi, 2002). One-dimensional structures, however, have only been reported in few cases for Ln_2_Cu complexes with the trichloroacetate ligand (Kutlu *et al.*,1997; Legendziewicz *et al.*, 2000)

Aliphatic monocarboxylic acids are expected to have limited bonding ability, since the carboxylate group cannot bridge adjacent large high-nuclear clusters. Monocarboxylates, however, may constitute a route toward one-dimensional 3 d-4f polymers, their main interest being the construction of single chain magnets. We recently used *trans*-2-butenoic acid in the synthesis and X-ray structure determination of three isostructural Cu^II^—Ln^III^1-D polymers {[Cu_2_Ln_2_*L*_10_(H_2_O)_4_].3H_2_O}_n_(Ln = Gd^III^, Er^III^, Y^III^; HL= *trans*-2-butenoicacid) (Calvo *et al.*, 2008). The {[Cu_2_Ho_2_*L*_10_(H_2_O)_4_].3H_2_O}_n_ species had also been synthesized, but no single crystals could be obtained at the time. We now wish to report the crystal data of the isostructural Cu_2_Ho_2 _complex obtained as well shaped needles. They consist of non-centrosymmetric polymeric chains built in the centrosymmetric *P*2_1_/*c* group by two distinct blocks: one formed by two Cu^II^ ions bridged by four carboxylate bridges in the η^1^:η^1^:µ_2_ conformation and the other by two Ho^III^ ions bridged by two carboxylate oxygen atoms in η^2^:η^1^:µ_2_ conformation (See Figure 1). Bonds between Cu and Ho metal centers consist of one covalent tridentate carboxylate oxygen atom and two intrachain H-bonds which enhance the stability of the chains.

Each Cu^II^ atom of the Cu_2 _dinuclear core provides the basal plane for a square pyramidal arrangement, the apical sites being provided by atoms O16 and O19 common to the Ho_2 _coordination polyhedra. The Cu_2_ block resembles the structure of dinuclear copper acetate monohydrate (Van Niekerk & Schoening, 1953), the main difference being the absence of an embedded inversion symmetry center relating the copper ions, present in the latter. The four independent carboxylato bridges in the Cu_2_ structure depart an average of 0.12 Å from a centrosymmetric disposition, as measured by the best fit of the dinuclear unit with its inverted image (*XP* in the *SHELXTL* package, Sheldrick, 2008). The four Cu—O—C—O—Cu loops are planar within 0.03 Å, and parallel or perpendicular to each other within a maximum deviation of *ca *6°. The Cu···Cu distance within the dinuclear unit is 2.642 (1) Å compared with 2.616 (1) Å in the classical fourfold copper acetate monohydrate. In the Ho_2_ block, the metal centers are bridged by two oxygen atoms from two tridentate η^2^:η^1^:µ_2_ carboxylates at a Ho···Ho distance of 4.207 (1) Å.The coordination of each holmium (HoO_9_) is completed by two chelating carboxylates and two aqua O atoms. Departure from a centrosymmetric arrangement is more important in this block than in the copper one, the mean square deviation from the inverted image being here 0.27 Å. Within a chain, Figure 2, alternate dinuclear units of the same type are related by a unit cell *a* translation; all the space group symmetry elements are external to the chains and thus relate them into each other. As a consequence of this lack of internal symmetry, alternate Cu—Ho chemical bridges are slightly different: 4.496 and 4.517 Å, respectively. The axis-to-axis separations between a chain and the six neighbouring ones cover the range 10–14 Å. The alkene groups protrude outwards almost normal to the chain direction. Although hydrogen atoms of water molecules could not be found in the late difference Fourier maps, a number of short Ow···OCO_2 _and Ow···Ow distances, less than 3.00 Å (Table 2), suggests involvement in H-bonding interactions

### Experimental

The title compound was prepared by the slow addition under constant stirring of Ho_2_(CO_3_)_3_(0.50 g,1 mmol) to an aqueous solution of copper acetate monohydrate (0.40 g, 2 mmol) and *trans*-2-butenoic acid (0.90 g, 10 mmol) at pH about5. The filtrate was stored in a stoppered flask for two months whereupon green needles of (I) were obtained (75% yield based on Cu). Anal Calcd(found) for C_40_H_64_O_27_Cu_2_Ho_2_: C, 33.80 (34.20), H, 4.50 (4.75), Cu, 8.95 (9.10)%.IR (KBr disk, cm^-1^): 3427(s, vbr, ν(OH)), 1659, 1603 and 1538(*versus*,ν(CO2)*asym*), 1449 and 1417(*versus*, ν(CO2)*sym*), 1297(*m*), 1256(*m*), 1105(w), 967(*m*),917(w), 857(w), 749(*m*), 699(w), 651(br, w), 521(w), 461(w), 420(w). The TGA showed that the water loss occurs in the range 80–112 °C (see refinement section for details); decomposition occurs in three overlapping steps, in the range 245–550°C.

### Refinement

Only seven water-molecule O atoms could be clearly found and refined: four of them bound to the metal centers and the remaining three as crystallization solvates. A *PLATON* check, however, detected voids capable to lodge further trapped solvent molecules and a SQUEEZE run gave a correction for some 44 delocalized electrons, roughly two and a half diffused water molecules. The final refinement was made against these corrected data.

On the other hand, a TGA run showed that the mass loss occurring in the range 353–385 K corresponded to about 8.5 water molecules per formula unit, leaving only one and a half molecules not accounted for. Even if not in strict accordance, both TGA and SQUEEZE thus coincide in the existence of a fraction of delocalized crystallization water, in the range 1.5–2.5 molecules per formula unit.

Hydrogen atoms attached to carbon were placed at calculated positions (C—H: 0.93–0.96 Å) and allowed to ride; methyl groups were allowed to rotate as well; displacement factors were taken as U(H)_iso_ = *x*.U_eq_(host), *x*: 1.2–1.5

The H atoms attached to water molecules could not be found in the difference Fourier and were accordingly disregarded from the final model. The final formula was stated in terms of the water molecules effectively refined.

Finally, some soft similarity restraints were introduced among similar bonds in the highly vibrating butenoate moieties, so as to assure meaningful C—C distances.

### Crystal data

**Table:**

[Cu_2_Ho_2_(C_4_H_5_O_2_)_10_(H_2_O)_4_]·3H_2_O	*F*(000) = 2792
*M**_r_* = 1433.85	*D*_x_ = 1.638 Mg m^−^^3^
Monoclinic, *P*2_1_/*c*	Mo *K*α radiation, λ = 0.71073 Å
Hall symbol: -P 2ybc	Cell parameters from 7221 reflections
*a* = 13.8856 (17) Å	θ = 2.1–26.8°
*b* = 22.078 (3) Å	µ = 3.49 mm^−^^1^
*c* = 19.846 (3) Å	*T* = 291 K
β = 107.152 (2)°	Needles, green
*V* = 5813.5 (14) Å^3^	0.24 × 0.10 × 0.08 mm
*Z* = 4	

### Data collection

**Table:**

Bruker SMART CCD area-detector diffractometer	12977 independent reflections
Radiation source: fine-focus sealed tube	9482 reflections with *I* > 2σ(*I*)
graphite	*R*_int_ = 0.043
φ and ω scans	θ_max_ = 28.2°, θ_min_ = 1.8°
Absorption correction: multi-scan (SADABS; (Bruker, 2002)	*h* = −18→13
*T*_min_ = 0.62, *T*_max_ = 0.76	*k* = −29→25
34338 measured reflections	*l* = −19→26

### Refinement

**Table:**

Refinement on *F*^2^	Primary atom site location: structure-invariant direct methods
Least-squares matrix: full	Secondary atom site location: difference Fourier map
*R*[*F*^2^ > 2σ(*F*^2^)] = 0.040	Hydrogen site location: inferred from neighbouring sites
*wR*(*F*^2^) = 0.085	H-atom parameters constrained
*S* = 0.93	*w* = 1/[σ^2^(*F*_o_^2^) + (0.0296*P*)^2^] where *P* = (*F*_o_^2^ + 2*F*_c_^2^)/3
12977 reflections	(Δ/σ)_max_ = 0.003
653 parameters	Δρ_max_ = 1.23 e Å^−^^3^
30 restraints	Δρ_min_ = −1.02 e Å^−^^3^

### Special details

**Table:**

Geometry. All e.s.d.'s (except the e.s.d. in the dihedral angle between two l.s. planes) are estimated using the full covariance matrix. The cell e.s.d.'s are taken into account individually in the estimation of e.s.d.'s in distances, angles and torsion angles; correlations between e.s.d.'s in cell parameters are only used when they are defined by crystal symmetry. An approximate (isotropic) treatment of cell e.s.d.'s is used for estimating e.s.d.'s involving l.s. planes.

### Fractional atomic coordinates and isotropic or equivalent isotropic displacement parameters (Å^2^)

**Table:**

	*x*	*y*	*z*	*U*_iso_*/*U*_eq_	
Ho1	0.843231 (18)	0.783533 (10)	0.670233 (12)	0.03660 (7)	
Ho2	1.024471 (17)	0.664760 (10)	0.594522 (11)	0.03346 (7)	
Cu1	0.36154 (5)	0.68897 (3)	0.65391 (3)	0.03839 (15)	
Cu2	0.51325 (5)	0.74316 (3)	0.62360 (3)	0.04014 (15)	
O11	0.3036 (3)	0.77154 (15)	0.64194 (18)	0.0456 (9)	
O21	0.4304 (3)	0.81524 (15)	0.61285 (19)	0.0513 (10)	
C11	0.3485 (4)	0.8172 (2)	0.6260 (3)	0.0403 (12)	
C21	0.2976 (5)	0.8760 (2)	0.6233 (3)	0.0546 (15)	
H21	0.2337	0.8768	0.6294	0.065*	
C31	0.3377 (6)	0.9269 (3)	0.6128 (4)	0.079 (2)	
H31	0.4007	0.9254	0.6053	0.095*	
C41	0.2900 (7)	0.9879 (3)	0.6120 (5)	0.130 (4)	
H411	0.2243	0.9831	0.6180	0.195*	
H412	0.2840	1.0075	0.5678	0.195*	
H413	0.3312	1.0122	0.6497	0.195*	
O12	0.4345 (3)	0.70699 (16)	0.75125 (18)	0.0511 (10)	
O22	0.5570 (3)	0.75996 (17)	0.72663 (18)	0.0515 (10)	
C12	0.5109 (4)	0.7396 (2)	0.7686 (3)	0.0417 (12)	
C22	0.5494 (4)	0.7565 (3)	0.8436 (3)	0.0545 (15)	
H22	0.5406	0.7287	0.8766	0.065*	
C32	0.5943 (4)	0.8065 (3)	0.8670 (3)	0.0617 (17)	
H32	0.6102	0.8320	0.8346	0.074*	
C42	0.6232 (5)	0.8273 (3)	0.9430 (3)	0.082 (2)	
H421	0.6212	0.7934	0.9729	0.123*	
H422	0.5767	0.8577	0.9484	0.123*	
H423	0.6901	0.8438	0.9559	0.123*	
O13	0.4443 (3)	0.61669 (15)	0.6601 (2)	0.0546 (10)	
O23	0.5778 (3)	0.66311 (15)	0.64296 (19)	0.0499 (9)	
C13	0.5324 (4)	0.6173 (2)	0.6572 (3)	0.0441 (13)	
C23	0.5903 (5)	0.5598 (2)	0.6700 (3)	0.0575 (16)	
H23	0.6581	0.5609	0.6723	0.069*	
C33	0.5505 (5)	0.5080 (3)	0.6783 (3)	0.075 (2)	
H33	0.4830	0.5079	0.6770	0.090*	
C43	0.6053 (6)	0.4482 (3)	0.6898 (4)	0.109 (3)	
H431	0.5809	0.4235	0.6485	0.164*	
H432	0.5938	0.4280	0.7296	0.164*	
H433	0.6763	0.4551	0.6987	0.164*	
O14	0.3123 (3)	0.67875 (17)	0.55130 (17)	0.0509 (10)	
O24	0.4430 (3)	0.72049 (16)	0.52530 (18)	0.0514 (10)	
C14	0.3585 (4)	0.6959 (2)	0.5083 (3)	0.0445 (13)	
C24	0.3035 (4)	0.6850 (3)	0.4335 (3)	0.0565 (16)	
H24	0.2503	0.6577	0.4237	0.068*	
C34	0.3231 (5)	0.7100 (3)	0.3815 (3)	0.0696 (19)	
H34	0.3779	0.7362	0.3915	0.084*	
C44	0.2657 (5)	0.7010 (3)	0.3048 (3)	0.084 (2)	
H441	0.2093	0.6747	0.3011	0.126*	
H442	0.3092	0.6831	0.2807	0.126*	
H443	0.2419	0.7394	0.2839	0.126*	
O15	0.9577 (3)	0.57490 (15)	0.52451 (18)	0.0459 (9)	
O25	0.8763 (3)	0.61044 (15)	0.59465 (19)	0.0471 (9)	
C15	0.8831 (4)	0.5728 (2)	0.5473 (3)	0.0428 (13)	
C25	0.8068 (5)	0.5250 (3)	0.5189 (3)	0.0639 (17)	
H25	0.8221	0.4948	0.4911	0.077*	
C35	0.7229 (5)	0.5231 (3)	0.5303 (3)	0.0727 (19)	
H35	0.7099	0.5522	0.5604	0.087*	
C45	0.6403 (5)	0.4769 (3)	0.4990 (4)	0.110 (3)	
H451	0.6616	0.4504	0.4678	0.164*	
H452	0.5799	0.4976	0.4733	0.164*	
H453	0.6275	0.4536	0.5363	0.164*	
O16	1.2173 (2)	0.65248 (14)	0.66304 (17)	0.0389 (8)	
O26	1.1059 (3)	0.58251 (14)	0.66393 (18)	0.0442 (9)	
C16	1.1965 (4)	0.6008 (2)	0.6824 (2)	0.0364 (11)	
C26	1.2771 (4)	0.5619 (2)	0.7272 (3)	0.0472 (14)	
H26	1.3425	0.5771	0.7414	0.057*	
C36	1.2615 (4)	0.5076 (2)	0.7477 (3)	0.0640 (18)	
H36	1.1953	0.4938	0.7347	0.077*	
C46	1.3410 (5)	0.4654 (3)	0.7906 (4)	0.094 (3)	
H461	1.4061	0.4839	0.7995	0.141*	
H462	1.3396	0.4283	0.7650	0.141*	
H463	1.3281	0.4568	0.8346	0.141*	
O17	0.8752 (3)	0.74373 (14)	0.57064 (16)	0.0402 (8)	
O27	0.9215 (3)	0.70443 (15)	0.48474 (17)	0.0448 (9)	
C17	0.8725 (4)	0.7441 (2)	0.5062 (3)	0.0383 (12)	
C27	0.8146 (4)	0.7912 (2)	0.4588 (3)	0.0461 (14)	
H27	0.7746	0.8172	0.4758	0.055*	
C37	0.8169 (5)	0.7980 (2)	0.3939 (3)	0.0523 (15)	
H37	0.8562	0.7712	0.3772	0.063*	
C47	0.7609 (6)	0.8460 (3)	0.3448 (3)	0.083 (2)	
H471	0.7123	0.8645	0.3641	0.124*	
H472	0.7268	0.8281	0.3000	0.124*	
H473	0.8074	0.8761	0.3387	0.124*	
O18	0.9594 (3)	0.75324 (16)	0.77741 (18)	0.0502 (10)	
O28	0.9992 (3)	0.69894 (16)	0.69837 (18)	0.0505 (10)	
C18	1.0119 (4)	0.7120 (2)	0.7621 (3)	0.0416 (13)	
C28	1.0866 (5)	0.6785 (3)	0.8176 (3)	0.0681 (19)	
H28	1.1244	0.6486	0.8042	0.082*	
C38	1.1021 (6)	0.6886 (4)	0.8835 (4)	0.104 (3)	
H38	1.0625	0.7179	0.8962	0.125*	
C48	1.1803 (8)	0.6563 (4)	0.9425 (4)	0.187 (6)	
H481	1.2200	0.6300	0.9227	0.281*	
H482	1.1469	0.6327	0.9697	0.281*	
H483	1.2233	0.6856	0.9725	0.281*	
O19	0.6491 (3)	0.78103 (15)	0.60660 (17)	0.0426 (9)	
O29	0.7445 (3)	0.85441 (15)	0.58965 (18)	0.0480 (9)	
C19	0.6600 (4)	0.8298 (2)	0.5752 (3)	0.0394 (12)	
C29	0.5717 (5)	0.8531 (3)	0.5191 (3)	0.0660 (18)	
H29	0.5109	0.8325	0.5110	0.079*	
C39	0.5738 (6)	0.8983 (3)	0.4821 (4)	0.109 (3)	
H39	0.6349	0.9185	0.4903	0.130*	
C49	0.4842 (7)	0.9230 (4)	0.4242 (5)	0.185 (5)	
H491	0.5080	0.9451	0.3906	0.277*	
H492	0.4457	0.9496	0.4447	0.277*	
H493	0.4424	0.8901	0.4010	0.277*	
O10	0.9850 (3)	0.83683 (15)	0.6588 (2)	0.0540 (10)	
O20	0.9038 (3)	0.87976 (15)	0.72448 (18)	0.0488 (9)	
C10	0.9735 (4)	0.8813 (2)	0.6968 (3)	0.0444 (13)	
C20	1.0431 (4)	0.9329 (2)	0.7057 (3)	0.0562 (16)	
H20	1.0967	0.9297	0.6867	0.067*	
C30	1.0347 (5)	0.9820 (3)	0.7378 (3)	0.0683 (18)	
H30	0.9817	0.9838	0.7575	0.082*	
C40	1.1009 (5)	1.0372 (3)	0.7473 (4)	0.094 (3)	
H401	1.1487	1.0322	0.7213	0.141*	
H402	1.1362	1.0424	0.7964	0.141*	
H403	1.0600	1.0723	0.7303	0.141*	
O1W	0.7526 (3)	0.80013 (15)	0.74993 (17)	0.0470 (9)	
O2W	0.7832 (3)	0.68213 (14)	0.66900 (18)	0.0445 (9)	
O3W	1.0957 (2)	0.76302 (14)	0.60445 (18)	0.0432 (9)	
O4W	1.1179 (3)	0.65241 (16)	0.51587 (17)	0.0474 (9)	
O5W	0.8129 (3)	0.86530 (16)	0.86586 (17)	0.0542 (10)	
O6W	0.9477 (3)	0.96277 (14)	0.88853 (18)	0.0541 (10)	
O7W	1.0534 (3)	0.85442 (18)	0.87360 (18)	0.0610 (11)	

### Atomic displacement parameters (Å^2^)

**Table:**

	*U*^11^	*U*^22^	*U*^33^	*U*^12^	*U*^13^	*U*^23^
Ho1	0.03854 (14)	0.04022 (14)	0.03143 (13)	−0.00543 (11)	0.01091 (10)	−0.00266 (10)
Ho2	0.03031 (13)	0.03573 (13)	0.03362 (13)	−0.00186 (10)	0.00829 (9)	−0.00210 (10)
Cu1	0.0296 (3)	0.0453 (4)	0.0392 (4)	−0.0049 (3)	0.0086 (3)	−0.0007 (3)
Cu2	0.0313 (4)	0.0485 (4)	0.0406 (4)	−0.0052 (3)	0.0106 (3)	−0.0008 (3)
O11	0.041 (2)	0.042 (2)	0.055 (2)	−0.0044 (17)	0.0167 (18)	0.0005 (17)
O21	0.048 (2)	0.045 (2)	0.069 (3)	−0.0020 (19)	0.028 (2)	0.0026 (19)
C11	0.041 (3)	0.042 (3)	0.034 (3)	−0.001 (3)	0.005 (2)	−0.002 (2)
C21	0.062 (4)	0.046 (3)	0.063 (4)	0.001 (3)	0.030 (3)	−0.003 (3)
C31	0.099 (6)	0.048 (4)	0.104 (6)	0.004 (4)	0.050 (5)	0.003 (4)
C41	0.186 (10)	0.043 (4)	0.196 (10)	0.028 (5)	0.109 (8)	0.028 (5)
O12	0.040 (2)	0.071 (3)	0.042 (2)	−0.011 (2)	0.0111 (18)	−0.0002 (19)
O22	0.041 (2)	0.071 (3)	0.043 (2)	−0.014 (2)	0.0136 (18)	−0.0120 (19)
C12	0.033 (3)	0.048 (3)	0.043 (3)	0.005 (3)	0.011 (2)	0.001 (3)
C22	0.040 (3)	0.077 (4)	0.044 (3)	−0.003 (3)	0.009 (3)	0.004 (3)
C32	0.042 (4)	0.083 (4)	0.062 (4)	−0.006 (3)	0.019 (3)	−0.017 (3)
C42	0.068 (5)	0.113 (6)	0.063 (4)	−0.013 (4)	0.018 (4)	−0.030 (4)
O13	0.044 (2)	0.044 (2)	0.081 (3)	0.0001 (19)	0.027 (2)	0.002 (2)
O23	0.039 (2)	0.046 (2)	0.064 (3)	−0.0019 (18)	0.0141 (19)	0.0024 (19)
C13	0.039 (3)	0.050 (3)	0.038 (3)	−0.002 (3)	0.004 (2)	−0.002 (3)
C23	0.053 (4)	0.051 (4)	0.067 (4)	0.011 (3)	0.016 (3)	0.003 (3)
C33	0.088 (6)	0.052 (4)	0.081 (5)	0.008 (4)	0.017 (4)	0.005 (4)
C43	0.148 (9)	0.056 (4)	0.116 (7)	0.026 (5)	0.027 (6)	0.012 (4)
O14	0.036 (2)	0.081 (3)	0.034 (2)	−0.016 (2)	0.0072 (16)	−0.0097 (19)
O24	0.044 (2)	0.069 (3)	0.042 (2)	−0.016 (2)	0.0150 (18)	−0.0040 (19)
C14	0.040 (3)	0.051 (3)	0.041 (3)	−0.001 (3)	0.010 (3)	−0.004 (3)
C24	0.045 (4)	0.079 (4)	0.046 (4)	−0.011 (3)	0.015 (3)	−0.003 (3)
C34	0.066 (5)	0.089 (5)	0.058 (4)	−0.008 (4)	0.024 (4)	0.003 (4)
C44	0.090 (6)	0.123 (6)	0.040 (4)	0.007 (5)	0.019 (4)	0.002 (4)
O15	0.042 (2)	0.043 (2)	0.049 (2)	−0.0077 (18)	0.0093 (18)	−0.0114 (17)
O25	0.040 (2)	0.045 (2)	0.060 (2)	−0.0052 (17)	0.0204 (18)	−0.0024 (18)
C15	0.039 (3)	0.039 (3)	0.045 (3)	−0.003 (3)	0.002 (3)	0.005 (2)
C25	0.045 (4)	0.060 (4)	0.079 (5)	−0.004 (3)	0.008 (3)	−0.011 (3)
C35	0.063 (5)	0.073 (5)	0.075 (5)	−0.012 (4)	0.010 (4)	−0.006 (4)
C45	0.084 (6)	0.090 (5)	0.136 (7)	−0.046 (5)	0.002 (5)	−0.020 (5)
O16	0.036 (2)	0.0381 (19)	0.042 (2)	−0.0043 (16)	0.0115 (16)	0.0031 (16)
O26	0.032 (2)	0.041 (2)	0.054 (2)	−0.0085 (16)	0.0024 (17)	0.0028 (17)
C16	0.038 (3)	0.037 (3)	0.033 (3)	−0.001 (2)	0.009 (2)	−0.004 (2)
C26	0.035 (3)	0.050 (3)	0.049 (3)	−0.006 (3)	0.001 (2)	0.007 (3)
C36	0.051 (4)	0.054 (4)	0.070 (4)	−0.013 (3)	−0.010 (3)	0.015 (3)
C46	0.088 (6)	0.057 (4)	0.105 (6)	0.003 (4)	−0.021 (5)	0.024 (4)
O17	0.049 (2)	0.043 (2)	0.0288 (18)	−0.0020 (17)	0.0107 (16)	−0.0016 (15)
O27	0.044 (2)	0.051 (2)	0.038 (2)	0.0139 (18)	0.0082 (17)	−0.0007 (16)
C17	0.038 (3)	0.039 (3)	0.035 (3)	−0.004 (2)	0.007 (2)	−0.001 (2)
C27	0.057 (4)	0.045 (3)	0.038 (3)	0.014 (3)	0.018 (3)	0.004 (2)
C37	0.072 (4)	0.043 (3)	0.044 (3)	0.007 (3)	0.020 (3)	0.003 (3)
C47	0.121 (7)	0.067 (4)	0.056 (4)	0.017 (4)	0.020 (4)	0.019 (3)
O18	0.047 (2)	0.059 (2)	0.042 (2)	0.006 (2)	0.0083 (18)	−0.0040 (18)
O28	0.056 (3)	0.061 (2)	0.038 (2)	−0.012 (2)	0.0182 (18)	−0.0082 (18)
C18	0.040 (3)	0.051 (3)	0.032 (3)	−0.013 (3)	0.007 (2)	0.002 (2)
C28	0.068 (5)	0.074 (4)	0.051 (4)	0.017 (4)	0.001 (3)	0.005 (3)
C38	0.120 (8)	0.117 (6)	0.053 (5)	0.005 (5)	−0.007 (5)	0.024 (5)
C48	0.213 (13)	0.177 (10)	0.102 (7)	0.000 (9)	−0.061 (7)	0.078 (7)
O19	0.039 (2)	0.048 (2)	0.042 (2)	−0.0068 (17)	0.0119 (16)	0.0040 (17)
O29	0.046 (2)	0.047 (2)	0.055 (2)	−0.0074 (18)	0.0206 (19)	0.0058 (18)
C19	0.042 (3)	0.043 (3)	0.036 (3)	0.002 (3)	0.016 (2)	−0.001 (2)
C29	0.054 (4)	0.086 (5)	0.056 (4)	0.011 (4)	0.015 (3)	0.025 (3)
C39	0.071 (6)	0.119 (7)	0.118 (7)	0.004 (5)	0.001 (5)	0.065 (6)
C49	0.118 (9)	0.217 (11)	0.181 (10)	0.038 (8)	−0.016 (7)	0.143 (9)
O10	0.057 (3)	0.045 (2)	0.069 (3)	−0.0125 (19)	0.032 (2)	−0.0162 (19)
O20	0.050 (2)	0.049 (2)	0.053 (2)	−0.0096 (19)	0.0251 (19)	−0.0109 (18)
C10	0.046 (3)	0.044 (3)	0.044 (3)	0.000 (3)	0.014 (3)	−0.004 (3)
C20	0.060 (4)	0.046 (3)	0.072 (4)	−0.015 (3)	0.034 (3)	−0.014 (3)
C30	0.068 (5)	0.063 (4)	0.083 (5)	−0.017 (4)	0.036 (4)	−0.021 (4)
C40	0.103 (6)	0.060 (4)	0.137 (7)	−0.037 (4)	0.061 (5)	−0.041 (4)
O1W	0.044 (2)	0.060 (2)	0.040 (2)	−0.0070 (18)	0.0162 (17)	−0.0091 (17)
O2W	0.036 (2)	0.044 (2)	0.056 (2)	0.0000 (17)	0.0175 (17)	−0.0007 (17)
O3W	0.038 (2)	0.0354 (18)	0.058 (2)	−0.0041 (16)	0.0184 (18)	−0.0023 (17)
O4W	0.033 (2)	0.074 (3)	0.037 (2)	−0.0042 (18)	0.0130 (16)	−0.0078 (18)
O5W	0.058 (3)	0.060 (2)	0.040 (2)	−0.013 (2)	0.0087 (18)	−0.0083 (18)
O6W	0.069 (3)	0.038 (2)	0.053 (2)	−0.0057 (19)	0.014 (2)	−0.0005 (17)
O7W	0.054 (3)	0.085 (3)	0.041 (2)	0.012 (2)	0.0101 (19)	0.002 (2)

### Geometric parameters (Å, °)

**Table:**

Ho1—O17	2.322 (3)	C34—C44	1.507 (7)
Ho1—O1W	2.323 (3)	C34—H34	0.9300
Ho1—O18	2.356 (3)	C44—H441	0.9600
Ho1—O10	2.362 (4)	C44—H442	0.9600
Ho1—O29	2.365 (3)	C44—H443	0.9600
Ho1—O2W	2.386 (3)	O15—C15	1.247 (6)
Ho1—O20	2.418 (3)	O25—C15	1.279 (6)
Ho1—O19	2.619 (3)	C15—C25	1.484 (7)
Ho1—C10	2.765 (5)	C25—C35	1.251 (7)
Ho1—O28	2.789 (4)	C25—H25	0.9300
Ho2—O28	2.316 (3)	C35—C45	1.523 (7)
Ho2—O4W	2.322 (3)	C35—H35	0.9300
Ho2—O26	2.357 (3)	C45—H451	0.9600
Ho2—O3W	2.368 (3)	C45—H452	0.9600
Ho2—O25	2.382 (3)	C45—H453	0.9600
Ho2—O27	2.392 (3)	O16—C16	1.265 (5)
Ho2—O15	2.443 (3)	O16—Cu1^ii^	2.215 (3)
Ho2—O16	2.635 (3)	O26—C16	1.267 (5)
Ho2—O17	2.642 (3)	C16—C26	1.481 (6)
Ho2—C17	2.901 (5)	C26—C36	1.305 (6)
Cu1—O12	1.939 (3)	C26—H26	0.9300
Cu1—O13	1.949 (4)	C36—C46	1.503 (7)
Cu1—O14	1.961 (3)	C36—H36	0.9300
Cu1—O11	1.978 (3)	C46—H461	0.9600
Cu1—O16^i^	2.215 (3)	C46—H462	0.9600
Cu1—Cu2	2.6417 (9)	C46—H463	0.9600
Cu2—O21	1.938 (4)	O17—C17	1.268 (5)
Cu2—O23	1.968 (3)	O27—C17	1.259 (5)
Cu2—O24	1.971 (3)	C17—C27	1.471 (6)
Cu2—O22	1.989 (3)	C27—C37	1.306 (6)
Cu2—O19	2.178 (3)	C27—H27	0.9300
O11—C11	1.272 (6)	C37—C47	1.493 (6)
O21—C11	1.241 (6)	C37—H37	0.9300
C11—C21	1.472 (6)	C47—H471	0.9600
C21—C31	1.297 (7)	C47—H472	0.9600
C21—H21	0.9300	C47—H473	0.9600
C31—C41	1.499 (7)	O18—C18	1.258 (6)
C31—H31	0.9300	O28—C18	1.258 (5)
C41—H411	0.9600	C18—C28	1.471 (7)
C41—H412	0.9600	C28—C38	1.282 (7)
C41—H413	0.9600	C28—H28	0.9300
O12—C12	1.244 (6)	C38—C48	1.520 (8)
O22—C12	1.273 (6)	C38—H38	0.9300
C12—C22	1.472 (6)	C48—H481	0.9600
C22—C32	1.286 (7)	C48—H482	0.9600
C22—H22	0.9300	C48—H483	0.9600
C32—C42	1.513 (7)	O19—C19	1.274 (5)
C32—H32	0.9300	O29—C19	1.247 (6)
C42—H421	0.9600	C19—C29	1.484 (7)
C42—H422	0.9600	C29—C39	1.244 (7)
C42—H423	0.9600	C29—H29	0.9300
O13—C13	1.241 (6)	C39—C49	1.524 (8)
O23—C13	1.268 (6)	C39—H39	0.9300
C13—C23	1.483 (6)	C49—H491	0.9600
C23—C33	1.302 (7)	C49—H492	0.9600
C23—H23	0.9300	C49—H493	0.9600
C33—C43	1.507 (7)	O10—C10	1.276 (5)
C33—H33	0.9300	O20—C10	1.247 (6)
C43—H431	0.9600	C10—C20	1.471 (6)
C43—H432	0.9600	C20—C30	1.280 (6)
C43—H433	0.9600	C20—H20	0.9300
O14—C14	1.268 (6)	C30—C40	1.504 (7)
O24—C14	1.246 (6)	C30—H30	0.9300
C14—C24	1.474 (6)	C40—H401	0.9600
C24—C34	1.269 (7)	C40—H402	0.9600
C24—H24	0.9300	C40—H403	0.9600
			
O17—Ho1—O1W	156.53 (12)	H421—C42—H423	109.5
O17—Ho1—O18	114.16 (12)	H422—C42—H423	109.5
O1W—Ho1—O18	78.12 (13)	C13—O13—Cu1	124.0 (4)
O17—Ho1—O10	74.47 (12)	C13—O23—Cu2	122.2 (3)
O1W—Ho1—O10	128.45 (12)	O13—C13—O23	125.5 (5)
O18—Ho1—O10	81.98 (13)	O13—C13—C23	118.4 (5)
O17—Ho1—O29	83.87 (12)	O23—C13—C23	116.2 (5)
O1W—Ho1—O29	91.91 (12)	C33—C23—C13	123.0 (6)
O18—Ho1—O29	154.75 (12)	C33—C23—H23	118.5
O10—Ho1—O29	86.49 (13)	C13—C23—H23	118.5
O17—Ho1—O2W	77.88 (11)	C23—C33—C43	125.1 (7)
O1W—Ho1—O2W	84.11 (12)	C23—C33—H33	117.5
O18—Ho1—O2W	83.50 (12)	C43—C33—H33	117.5
O10—Ho1—O2W	139.90 (12)	C33—C43—H431	109.5
O29—Ho1—O2W	118.86 (12)	C33—C43—H432	109.5
O17—Ho1—O20	125.65 (12)	H431—C43—H432	109.5
O1W—Ho1—O20	75.24 (12)	C33—C43—H433	109.5
O18—Ho1—O20	77.99 (13)	H431—C43—H433	109.5
O10—Ho1—O20	54.19 (12)	H432—C43—H433	109.5
O29—Ho1—O20	77.04 (12)	C14—O14—Cu1	125.1 (3)
O2W—Ho1—O20	154.61 (12)	C14—O24—Cu2	122.1 (3)
O17—Ho1—O19	90.55 (11)	O24—C14—O14	124.8 (5)
O1W—Ho1—O19	69.15 (11)	O24—C14—C24	120.7 (5)
O18—Ho1—O19	139.70 (12)	O14—C14—C24	114.5 (5)
O10—Ho1—O19	137.13 (12)	C34—C24—C14	125.3 (6)
O29—Ho1—O19	51.59 (11)	C34—C24—H24	117.4
O2W—Ho1—O19	70.65 (11)	C14—C24—H24	117.4
O20—Ho1—O19	113.99 (12)	C24—C34—C44	126.0 (6)
O17—Ho1—C10	100.30 (14)	C24—C34—H34	117.0
O1W—Ho1—C10	101.70 (14)	C44—C34—H34	117.0
O18—Ho1—C10	79.30 (14)	C34—C44—H441	109.5
O10—Ho1—C10	27.41 (13)	C34—C44—H442	109.5
O29—Ho1—C10	80.15 (14)	H441—C44—H442	109.5
O2W—Ho1—C10	160.21 (14)	C34—C44—H443	109.5
O20—Ho1—C10	26.79 (13)	H441—C44—H443	109.5
O19—Ho1—C10	129.13 (13)	H442—C44—H443	109.5
O17—Ho1—O28	65.47 (11)	C15—O15—Ho2	92.3 (3)
O1W—Ho1—O28	120.94 (11)	C15—O25—Ho2	94.3 (3)
O18—Ho1—O28	49.06 (11)	O15—C15—O25	119.4 (5)
O10—Ho1—O28	74.49 (12)	O15—C15—C25	117.7 (5)
O29—Ho1—O28	147.11 (11)	O25—C15—C25	122.8 (5)
O2W—Ho1—O28	67.79 (11)	C35—C25—C15	123.7 (6)
O20—Ho1—O28	110.60 (12)	C35—C25—H25	118.2
O19—Ho1—O28	135.29 (10)	C15—C25—H25	118.2
C10—Ho1—O28	93.36 (14)	C25—C35—C45	125.3 (7)
O28—Ho2—O4W	153.86 (12)	C25—C35—H35	117.4
O28—Ho2—O26	84.46 (12)	C45—C35—H35	117.4
O4W—Ho2—O26	92.03 (12)	C35—C45—H451	109.5
O28—Ho2—O3W	78.27 (12)	C35—C45—H452	109.5
O4W—Ho2—O3W	82.01 (12)	H451—C45—H452	109.5
O26—Ho2—O3W	122.71 (11)	C35—C45—H453	109.5
O28—Ho2—O25	78.79 (12)	H451—C45—H453	109.5
O4W—Ho2—O25	126.57 (12)	H452—C45—H453	109.5
O26—Ho2—O25	82.95 (12)	C16—O16—Cu1^ii^	130.2 (3)
O3W—Ho2—O25	143.21 (12)	C16—O16—Ho2	87.8 (3)
O28—Ho2—O27	118.99 (12)	Cu1^ii^—O16—Ho2	136.55 (14)
O4W—Ho2—O27	74.51 (12)	C16—O26—Ho2	100.8 (3)
O26—Ho2—O27	150.39 (11)	O16—C16—O26	119.7 (4)
O3W—Ho2—O27	82.13 (12)	O16—C16—C26	120.3 (5)
O25—Ho2—O27	84.32 (12)	O26—C16—C26	120.0 (4)
O28—Ho2—O15	129.71 (12)	C36—C26—C16	123.6 (5)
O4W—Ho2—O15	73.50 (12)	C36—C26—H26	118.2
O26—Ho2—O15	75.11 (11)	C16—C26—H26	118.2
O3W—Ho2—O15	150.52 (12)	C26—C36—C46	125.8 (6)
O25—Ho2—O15	53.73 (12)	C26—C36—H36	117.1
O27—Ho2—O15	75.79 (12)	C46—C36—H36	117.1
O28—Ho2—O16	88.38 (12)	C36—C46—H461	109.5
O4W—Ho2—O16	69.54 (11)	C36—C46—H462	109.5
O26—Ho2—O16	51.66 (10)	H461—C46—H462	109.5
O3W—Ho2—O16	73.46 (11)	C36—C46—H463	109.5
O25—Ho2—O16	133.95 (11)	H461—C46—H463	109.5
O27—Ho2—O16	138.68 (11)	H462—C46—H463	109.5
O15—Ho2—O16	111.57 (11)	C17—O17—Ho1	154.1 (3)
O28—Ho2—O17	68.17 (12)	C17—O17—Ho2	88.5 (3)
O4W—Ho2—O17	121.43 (11)	Ho1—O17—Ho2	115.70 (12)
O26—Ho2—O17	146.01 (11)	C17—O27—Ho2	100.6 (3)
O3W—Ho2—O17	72.35 (11)	O27—C17—O17	118.6 (4)
O25—Ho2—O17	72.57 (11)	O27—C17—C27	121.5 (4)
O27—Ho2—O17	50.84 (10)	O17—C17—C27	119.9 (5)
O15—Ho2—O17	106.72 (11)	O27—C17—Ho2	54.1 (2)
O16—Ho2—O17	141.66 (10)	O17—C17—Ho2	65.6 (3)
O28—Ho2—C17	93.83 (14)	C27—C17—Ho2	167.4 (4)
O4W—Ho2—C17	96.66 (13)	C37—C27—C17	123.0 (5)
O26—Ho2—C17	163.08 (13)	C37—C27—H27	118.5
O3W—Ho2—C17	73.06 (12)	C17—C27—H27	118.5
O25—Ho2—C17	80.22 (13)	C27—C37—C47	124.5 (6)
O27—Ho2—C17	25.24 (11)	C27—C37—H37	117.7
O15—Ho2—C17	93.46 (13)	C47—C37—H37	117.7
O16—Ho2—C17	145.23 (12)	C37—C47—H471	109.5
O17—Ho2—C17	25.92 (11)	C37—C47—H472	109.5
O12—Cu1—O13	88.88 (16)	H471—C47—H472	109.5
O12—Cu1—O14	167.90 (15)	C37—C47—H473	109.5
O13—Cu1—O14	89.40 (16)	H471—C47—H473	109.5
O12—Cu1—O11	90.28 (15)	H472—C47—H473	109.5
O13—Cu1—O11	166.73 (15)	C18—O18—Ho1	106.2 (3)
O14—Cu1—O11	88.65 (15)	C18—O28—Ho2	163.0 (4)
O12—Cu1—O16^i^	102.25 (14)	C18—O28—Ho1	85.3 (3)
O13—Cu1—O16^i^	103.16 (14)	Ho2—O28—Ho1	110.63 (13)
O14—Cu1—O16^i^	89.80 (14)	O18—C18—O28	119.3 (5)
O11—Cu1—O16^i^	89.96 (13)	O18—C18—C28	121.0 (5)
O12—Cu1—Cu2	85.23 (11)	O28—C18—C28	119.7 (5)
O13—Cu1—Cu2	83.69 (11)	C38—C28—C18	123.1 (7)
O14—Cu1—Cu2	82.68 (11)	C38—C28—H28	118.5
O11—Cu1—Cu2	83.04 (11)	C18—C28—H28	118.5
O16^i^—Cu1—Cu2	169.82 (9)	C28—C38—C48	124.9 (9)
O21—Cu2—O23	168.65 (16)	C28—C38—H38	117.6
O21—Cu2—O24	89.72 (16)	C48—C38—H38	117.6
O23—Cu2—O24	91.67 (15)	C38—C48—H481	109.5
O21—Cu2—O22	87.21 (16)	C38—C48—H482	109.5
O23—Cu2—O22	89.05 (15)	H481—C48—H482	109.5
O24—Cu2—O22	167.66 (15)	C38—C48—H483	109.5
O21—Cu2—O19	100.40 (14)	H481—C48—H483	109.5
O23—Cu2—O19	90.55 (14)	H482—C48—H483	109.5
O24—Cu2—O19	98.19 (14)	C19—O19—Cu2	129.3 (3)
O22—Cu2—O19	94.12 (14)	C19—O19—Ho1	87.9 (3)
O21—Cu2—Cu1	84.83 (11)	Cu2—O19—Ho1	138.47 (15)
O23—Cu2—Cu1	84.07 (11)	C19—O29—Ho1	100.7 (3)
O24—Cu2—Cu1	85.14 (11)	O29—C19—O19	119.7 (5)
O22—Cu2—Cu1	82.68 (11)	O29—C19—C29	122.2 (5)
O19—Cu2—Cu1	173.77 (9)	O19—C19—C29	117.9 (5)
C11—O11—Cu1	123.2 (3)	C39—C29—C19	124.3 (7)
C11—O21—Cu2	123.8 (3)	C39—C29—H29	117.9
O21—C11—O11	124.9 (5)	C19—C29—H29	117.9
O21—C11—C21	118.8 (5)	C29—C39—C49	125.2 (8)
O11—C11—C21	116.3 (5)	C29—C39—H39	117.4
C31—C21—C11	123.1 (6)	C49—C39—H39	117.4
C31—C21—H21	118.4	C39—C49—H491	109.5
C11—C21—H21	118.4	C39—C49—H492	109.5
C21—C31—C41	124.8 (7)	H491—C49—H492	109.5
C21—C31—H31	117.6	C39—C49—H493	109.5
C41—C31—H31	117.6	H491—C49—H493	109.5
C31—C41—H411	109.5	H492—C49—H493	109.5
C31—C41—H412	109.5	C10—O10—Ho1	94.2 (3)
H411—C41—H412	109.5	C10—O20—Ho1	92.3 (3)
C31—C41—H413	109.5	O20—C10—O10	119.3 (5)
H411—C41—H413	109.5	O20—C10—C20	122.4 (5)
H412—C41—H413	109.5	O10—C10—C20	118.3 (5)
C12—O12—Cu1	123.2 (3)	O20—C10—Ho1	60.9 (3)
C12—O22—Cu2	123.2 (3)	O10—C10—Ho1	58.4 (3)
O12—C12—O22	125.1 (5)	C20—C10—Ho1	175.9 (4)
O12—C12—C22	116.9 (5)	C30—C20—C10	124.3 (6)
O22—C12—C22	118.0 (5)	C30—C20—H20	117.9
C32—C22—C12	125.1 (6)	C10—C20—H20	117.9
C32—C22—H22	117.4	C20—C30—C40	127.3 (6)
C12—C22—H22	117.4	C20—C30—H30	116.3
C22—C32—C42	125.4 (6)	C40—C30—H30	116.3
C22—C32—H32	117.3	C30—C40—H401	109.5
C42—C32—H32	117.3	C30—C40—H402	109.5
C32—C42—H421	109.5	H401—C40—H402	109.5
C32—C42—H422	109.5	C30—C40—H403	109.5
H421—C42—H422	109.5	H401—C40—H403	109.5
C32—C42—H423	109.5	H402—C40—H403	109.5

Symmetry codes: (i) *x*−1, *y*, *z*; (ii) *x*+1, *y*, *z*.

### Table 2 Short OW···O contacts (< 3.00 Å) attributable to hydrogen bonding

**Table:**

OW···O	d (Å)		OW···O	d (Å)
O1W···O18	2.948 (5)		O3W···O28	2.957 (5)
O1W···O19	2.815 (5)		O4W···O14^i^	2.645 (5)
O1W···O20	2.895 (5)		O4W···O15	2.853 (5)
O1W···O22	2.763 (5)		O4W···O16	2.839 (4)
O1W···O5W	2.632 (5)		O4W···O27	2.854 (5)
O2W···O19	2.900 (5)		O4W···O7W^ii^	2.703 (5)
O2W···O23	2.777 (5)		O5W···O27^iii^	2.848 (5)
O2W···O25	2.734 (5)		O5W···O6W	2.800 (5)
O2W···O28	2.905 (5)		O6W···O15^iii^	2.788 (5)
O3W···O10	2.675 (5)		O6W···O26^iv^	2.858 (5)
O3W···O11^i^	2.766 (5)		O6W···O7W	2.867 (5)

Symmetry codes: (i) x+1, y, z; (ii) x, -y+3/2, z-1/2; (iii) x, -y+3/2, z+1/2; (iv) -x+2, y+1/2, -z+3/2.
